# Engaging with selective dry cow therapy: understanding the barriers and facilitators perceived by Irish farmers

**DOI:** 10.1186/s13620-021-00207-0

**Published:** 2021-10-23

**Authors:** Sarah Huey, Michaela Kavanagh, Aine Regan, Moira Dean, Clare McKernan, Finola McCoy, Eoin G. Ryan, Javier Caballero-Villalobos, Catherine I. McAloon

**Affiliations:** 1 Institute for Global Food Security, School of Biological Sciences, Queen’s University Belfast, Belfast, Northern Ireland; 2 Section of Herd Health and Animal Husbandry, School of Veterinary Medicine, University College Dublin, Belfield, Dublin 4 Ireland; 3 Agrifood Business and Spatial Analysis, Rural Economy Development Programme, Teagasc Mellows Campus, Athenry, Co. Galway Ireland; 4 Animal Health Ireland, 4-5 The Archways, Carrick on Shannon, Co. Leitrim N41 WN27 Ireland; 5 Department of Animal Production, Universidad de Córdoba, 14071 Córdoba, Spain

**Keywords:** Selective dry cow therapy, Farmer, Psychology, Behaviour change, CellCheck

## Abstract

**Background:**

Selective dry cow therapy (SDCT) is widely promoted in dairy farming as a method to reduce antimicrobial usage. New legislation introduced by the European Union will restrict and regulate the prophylactic and metaphylactic use of antibiotics from January 2022. Blanket dry cow therapy continues to be a practice engaged in by many farmers in Ireland and for many of these farmers, moving towards SDCT would require a significant infrastructural, behavioural and/or cultural change on their farm. Existing research has reported the important need to understand farmers’ motivations to initiate any substantial behaviour change. However, it is currently unknown what farmers know, think and believe about SDCT in Ireland. The aim of this study was to use qualitative methods to explore what barriers and facilitators farmers perceived to exist with SDCT and explore if they had chosen to implement SDCT after voluntarily participating in a funded dry cow consult with a trained veterinarian, with the objective of maximising the dry period udder health performance and moving safely to SDCT.

**Results:**

In this study, 19 farmers were contacted, and telephone interviews were conducted regarding farmers’ beliefs about the consequences of SDCT. Audio recordings were professionally transcribed verbatim and analysed qualitatively using an inductive thematic analysis. The analysis identified 6 barriers and 6 facilitators to implementing SDCT. A significant fear of increasing mastitis incidence was evident that caused reluctance towards SDCT and reliance on antibiotics. Mixed perceptions on SDCT, infrastructure limitations, a perceived lack of preventive advice as well as peer influence were presented as barriers to SDCT. Farmers can build confidence when a graded approach to SDCT is implemented, which could help overcome the fear of SDCT and reliance on antibiotics. Regulatory pressure, high standards of farm hygiene and use of targeted veterinary consults were found to facilitate SDCT. Education was suggested to motivate farmers in the future uptake of SDCT. Despite cited negative influences, peer influence can be utilised to encourage the farming community.

**Conclusions:**

This study prioritises areas to facilitate the major behaviour change required as a dairy industry in order to move from blanket dry cow therapy to SDCT.

**Supplementary Information:**

The online version contains supplementary material available at 10.1186/s13620-021-00207-0.

## Background

**Fig. 1:**
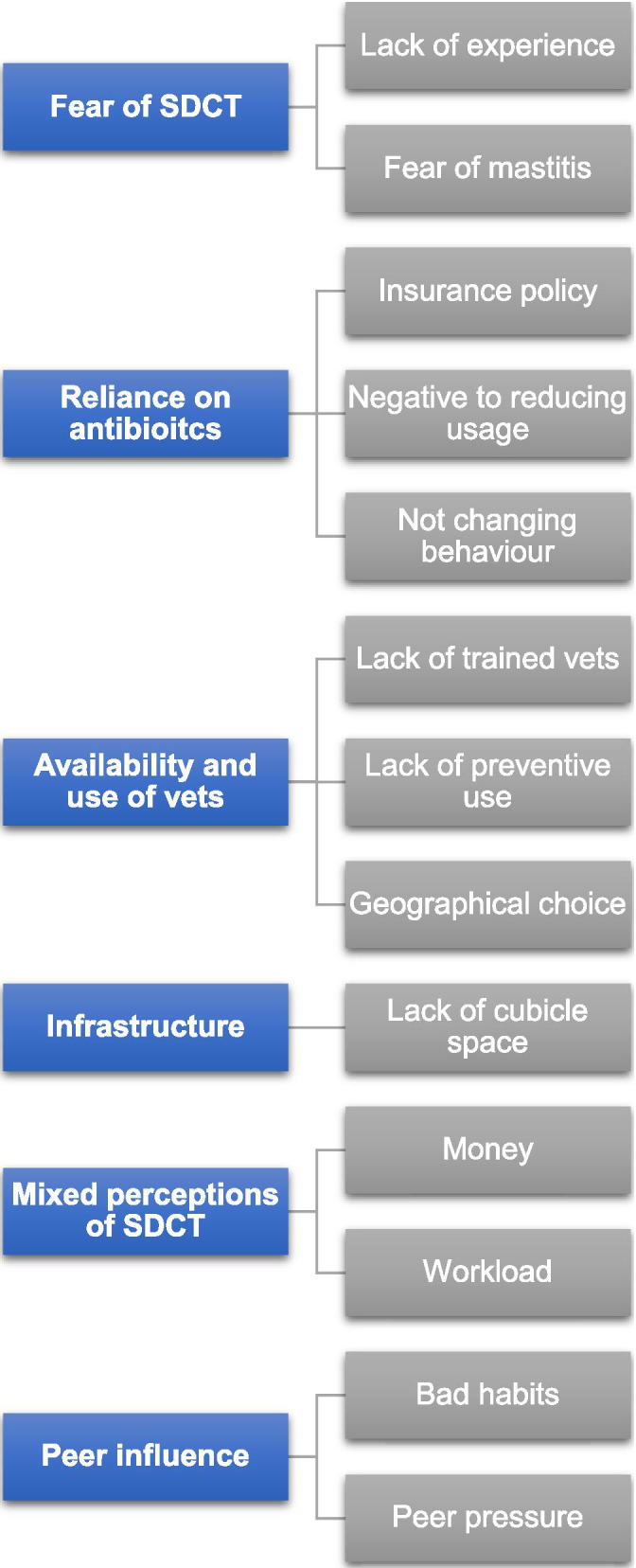
Perceived barriers identfied to selective dry cow therapy from interviewees

**Fig. 2:**
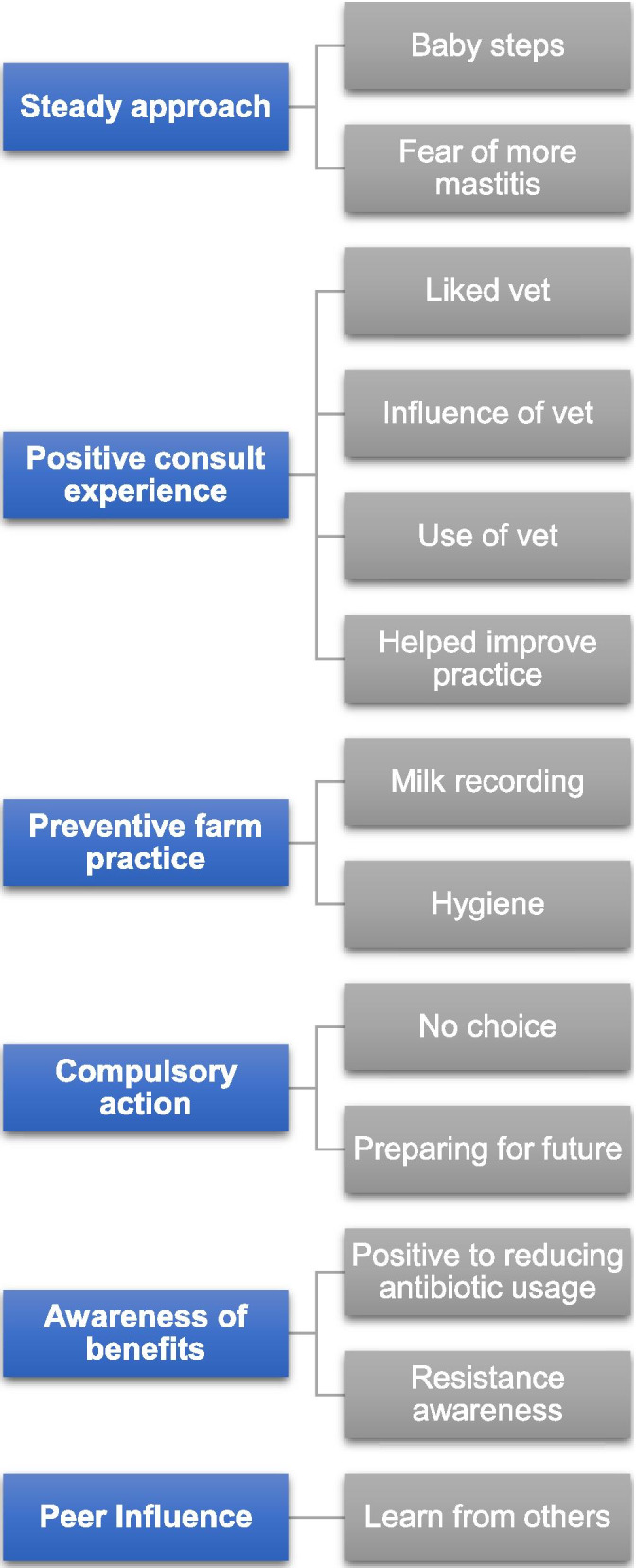
Facilitators to selective dry cow therapy identified from interviewees

Antimicrobial resistance (AMR) is a globally recognised public health issue that threatens the future of human and veterinary medical treatment [1, 2]. It is widely accepted that increased use of antibiotics in human and veterinary medicine accelerates the development of AMR and that increased use of antibiotics correlates with increased development of resistance [3, 4]. Internationally, a co-ordinated one health approach is being taken to address the AMR crisis [2] and the World Health Organization (WHO) have developed a global action plan to target AMR [5]. In response to the WHO global call to optimise the use of antimicrobials in human and veterinary medicine the European Union have introduced new regulations on veterinary medicines in Europe (Regulation EU 2019/6) which are due to come into effect in January 2022, which seek to harmonise the use of veterinary medicinal products across Europe. A main aim of the new legislation is to prohibit the preventive use of antibiotics in food-producing animals. This legislation is an important springboard and frames a major change in the prescribing of antibiotics for food producing animals [6] (Figs. 1 and 2).

The prophylactic antibiotic treatment of every cow when drying off, known as blanket dry cow therapy (BDCT), was historically widely adopted and promoted as a key pillar in mastitis control [7]. The objective of BDCT was to cure present infections and prevent new infections over the dry period [8]. However, the blanket approach implies that antibiotic dry cow therapy is given to all cows, including non-infected cows at drying off, and, therefore, BDCT is linked to the threat of AMR [9, 10]. An alternative strategy that promotes lower antimicrobial usage (AMU) is selective dry cow therapy (SDCT), where only cows with mastitis receive antibiotic treatment. In addition, some countries promote the use of a teat sealant, applied to all cows to prevent infection and only cows likely to contract mastitis receive antibiotic as part of the SDCT strategy [11]. This judgment on what cows receive antibiotic, is usually based on the cow’s somatic cell count (SCC) and mastitis records [8]. Adapting from BDCT to SDCT involves a new approach in terms of milk recording, time and technique of treatment which can affect SDCT implementation [11]. A study by More et al. [12] reported that, from sales data, enough dry cow intramammary antibiotic was sold to cover over 100 % of the national herd in 2015. More recent sales data also reports that dry cow antimicrobial sales remained close to coverage of 100 % of the national herd in 2019 again using sales data (personal communication), therefore BDCT is the norm in Ireland. Although widely practiced in other countries such as The Netherlands and Nordic countries [13], the practice of SDCT is not widely adopted in Ireland. Given that BDCT is habitually and culturally ingrained on many Irish farms, the transition towards SDCT will require significant behaviour change at an individual and industry level. On-going work within the Irish dairy industry is aiming to support this behaviour change.

CellCheck, a national mastitis control programme was formed in Ireland in 2010 and provides key resources for the optimisation of milk quality in the Irish dairy industry [12]. The CellCheck Technical Working Group have produced guidelines for farmers and veterinarians regarding a move towards selective dry cow therapy and revising the use of intramammary antibiotics on a preventative basis, as well as guidelines around use of the highest priority critically important antibiotics [14]. CellCheck is responsible for facilitating the industry to move towards a reduction in intramammary antibiotic use. There is an opportunity to reduce AMU in the national herd by increasing SDCT uptake. However, this requires a thorough understanding of farmers’ motivations to implement practices aimed at reducing AMU [15].

Awareness of and concern about AMR are not strong motivational drivers for AMU change at farm level; this is a finding which appears consistent across the literature [16]. In New Zealand, McDougall et al. [17], found that while farmers understood that there was a risk of AMR occurring on dairy farms, they did not agree that their use of antimicrobials was associated with the risk of AMR in human populations, or on other farms. Similarly, in a study with UK farmers, participants were more motivated to reduce AMU due to beliefs about cost reduction rather than concerns about the societal impact of AMR [18]. There is a need to expand the study of farmers’ motivations beyond beliefs about AMR and consider specific psychological constructs which are likely to influence behaviour change, such as individual beliefs about the practice in question, and the individual’s perception of their social, physical and cultural environment [19–21].

There has been limited research exploring what Irish farmers know and think about SDCT, however, research in other countries has demonstrated the importance of psychological constructs in determining farmers’ mastitis management practices. A psychological construct is a theoretical variable which provides an identifying name and a definition describing the aspects of human behaviour which it is depicting. They are useful as they allow us to build theory and predict what factors may determine human behaviour. Constructs explored in previous literature have included beliefs about consequences; attitudes, social norms; and perceived resource constraints [16, 22, 23]. This limited but growing body of literature indicates that individual beliefs and attitudes held by farmers specifically relating to the practice of SDCT are likely to be strong determinants of behaviour. For example, farmers’ beliefs about the consequences of transitioning towards SDCT appear to play a determining role in their actions. In a survey of farmers in The Netherlands, Scherpenzeel et al. [16] found that those farmers who held stronger negative beliefs about the consequences of SDCT and AMU were less likely to engage in the practice of SDCT. Similarly, a survey of British farmers found that 55% of those surveyed feared implementing SDCT as it could result in more death or mastitis [11]. Based on the emerging research in this area, specific psychological constructs are likely to play a defining role in influencing farmers’ (non-)engagement in SDCT. Prior to the development of any predictive theoretical models which could be used to inform future behaviour change interventions., exploratory qualitative research is first required to understand the nature and influence of possible psychological constructs shaping farmers’ (non-)engagement with SDCT. There has been no research exploring what farmers know, think and believe about SDCT in Ireland; a country in which SDCT engagement levels are much lower than some European and international counterparts [12]. Therefore, the aims of this study were to use inductive qualitative methods to elicit dairy farmers’ to talk about what they perceived to be barriers and facilitators of engaging with SDCT and explore what psychological constructs may be playing a part in shaping their behaviour.

## Results

From the themes, 6 key barriers were identified: (1) fear of SDCT; (2) reliance on antibiotics; (3) availability and use of veterinarians; (4) infrastructure; (5) mixed perceptions of SDCT and (6) peer influence. 6 key facilitators were also identified: (1) steady approach; (2) positive consult experience; (3) preventive farm practice; (4) compulsory action; (5) awareness of benefits to reducing AMU and (6) peer influence. Results are presented in Figs. 1 and 2. 

### Barriers to implementing SDCT

#### Fear of SDCT

In this theme, responses highlighted that many farmers were scared to make changes to their AMU for fear of increasing mastitis levels or “*fear of ruining what I have”.* These participants voiced they were the ones “*taking all the risk*”. There appeared to be a lack of confidence to try SDCT and farmers wanted to put off trying it:

Farmer 5: *“I think I’ll leave it another while...I still wouldn’t be confident enough yet”*

Farmer 15*: “No I think I’ll stick with the same I mean it worked last year so there’s no point in putting any pressure on them”*

#### Reliance on antibiotics

It became apparent that farmers had the mindset that BDCT provides them with the *“insurance”* of preventing mastitis and therefore are reluctant to lower their AMU. This mentality highlighted the beliefs which need to be overcome to introduce SDCT:

Farmer 2: *“People would be a bit hesitant about going away completely as they still have it as a kind of comfort blanket or insurance policy”*

Farmer 5: *“I wouldn’t be comfortable because it gives you peace of mind when they’re in the shed they’re sealed and there’s a cepravin [antibiotic] in them and that’s good enough, peace of mind totally”*

In some cases, there was a clear unwillingness to reduce AMU amongst farmers:

Farmer 11: “*While I’m allowed to do it, I’ll continue to do it...I wasn’t going to give myself any more extra work”*

Certain participants negatively recalled the public’s perception of reducing AMU:

Farmer 10: *“People have to understand that its actually harder to do this [drying off] properly than do it with an antibiotic”*

#### Availability and use of veterinarians

The majority of participants voiced that the main reason their veterinarian was chosen, was geographical proximity along with an apparent lack of trained veterinarians in their areas:

Farmer 1: *“There’s not an awful lot of them in our area that are qualified…. I was very sheepish and so I geographically picked who was next to me”*

Farmer 16: *“The more veterinarians that get involved in it the better rather than just maybe having the same few*”

There were few responses that acknowledged how they utilise their veterinarian normally, however some participants stated their views on how the curative use of veterinarians in farming needs changing:

Farmer 20*: “I would have a theory anyway that veterinarians should be going into farms for you know, prevention and they should be going into herds with advice on managing stuff instead of being fire brigades”*

#### Infrastructure

Along with high acknowledgement for hygiene, many responses highlighted how lacking cubicle space or land limited their ability to implement SDCT effectively due to an increased risk of infection:

Farmer 11: *“It’s an issue in every yard you go into now is that you don’t have enough cubicle space for animals”*

Farmer 16: “*Oh there was like you know housing definitely there are guys out there maybe with poor housing or maybe not enough cubicles or maybe not paying enough attention, selective dry cow probably won’t be for those unless they really get their act together”*

#### Mixed perceptions of SDCT

Mixed perceptions amongst farmers highlighted possible confusion that could affect SDCT implementation. In terms of finance, some responses voiced SDCT as a method of *“saving money”* whereas others disagreed:

Farmer 10: *“Well I’ll tell you I heard one lad saying, ‘I’m going to do that because it will be cheaper’ and that is just the totally wrong attitude because it’s not cheaper. When you take in your time, you take in your lime and stuff like that it would probably be cheaper to just bang in antibiotics”*

There were also negative perceptions on the workload involved with SDCT:

Farmer 13: *“Exactly, while they keep educating ye we’ll never get it all right because you’ll keep coming up with brainwaves to make our lives more difficult…when you’re the one in the pit in the morning it isn’t always as simple as it looks”*

#### Peer influence

This theme was widely cited by farmers as having an influence on their behaviour. As a barrier, responses highlighted how peer pressure can make farmers pick up *“bad habits*” from their peers or “*scare off*” others from introducing SDCT:

Farmer 1: *“The biggest thing farmers do is lie to one another and I feel that the peer pressure that comes in discussion groups to do something that is the trendy thing to be doing is a gripe for an old man like me”*

Farmer 5: *“I wouldn’t have done it on my own and I would’ve gone and gotten another farmer that was doing it and he probably would have given me bad practices”*

### Facilitators to implementing SDCT

#### Graded approach

During the discussion, it became evident participants who implemented SDCT did not want to vastly increase cow numbers. For many of the farmers interviewed, this was a new process that was *“scary at the outset”,* and there was a hesitancy to starting SDCT for fear of increasing mastitis cases. Farmers often referred to a *“baby step”* approach that they felt more comfortable with to build their confidence over time:

Farmer 16: *“But definitely start small, like with a certain percentage of your herd and like I did start small and do the small things right and it will work”*

Farmer 19: *“That’s the thing like you build your confidence with it and you’ll have more confidence with it next year”*

#### Positive consult experience

The majority of participants positively reviewed the consult with the veterinarian, with all farmers stating they would recommend it. The value of receiving advice from someone who takes a “*new perspective”* on their farm practice was acknowledged:

Farmer 4: *“Yeah I think somebody coming in from the outside just looking things is probably always a good thing no matter what you’re doing, somebody with a different view or different eyes on sides of what’s going on”*

Participants stated getting advice and *“small little tips”* from the veterinarian made the consult worthwhile, even if they were not implementing SDCT. It became evident that the most effective consults were when both parties contributed their ideas:

Farmer 17: *“Well I liked that she didn’t just say ‘yeah ok that’s fine’. We had a good old row about it like I didn’t just agree with her and she didn’t agree with me. We kept coming up with reasons for what we were doing and why we were doing it”*

The potential influence of the veterinarian in SDCT uptake was apparent from farmers expressing the importance of the veterinarian in future consults:

Farmer 18: *“I’d actually think that going forward that farmers should be using their veterinarians a bit more for consultations that way and procedures in place”*

#### Preventative farm practice

Across all participants, the importance of carrying out effective hygiene and good husbandry procedures on the farm was highlighted in order to prevent mastitis and reduce the need for antibiotics:

Farmer 16: *“Of course you know I think a bit of attention to detail maybe afterwards like your cubicles and your management of her afterwards. You can’t just hammer up sealer only and expect everything to be ok. Hygiene has to be crucial you know”*

Another important farm practice that aids the effective implementation of SDCT is regular milk recording and recording of mastitis cases, as stated:

Farmer 10: *“The records are the most important thing because you’re at nothing doing this thing if you haven’t got the records…..the lack of recording is a big thing with me I’m desperate you know if you don’t have a record how can you treat something? If you don’t know what the problem is, we can’t fix it”*

#### Compulsory action

When asked why they got involved in the consult, every participant gave a response indicating how SDCT was going to be compulsory soon and they were taking action. There was a mix of responses implying either a *“being imposed on us anyway”* attitude:

Farmer 7: *“I had to go through the process, so I went through the process*”

Or farmers expressed a want to get *“ahead of the game”* and be prepared for the future:

Farmer 16: *“I suppose I wanted to get in ahead of the posse and it’s coming down the line and at the end of the day someday we’ll have no other choice in the matter so I just wanted to get into it and I suppose get a feel for it before someone came and said its happening next year you know”*

#### Awareness of benefits to reducing antimicrobial usage

Although it was not always a reason cited, participants that recognized AMR as a priority were more accepting of SDCT as a means to reduce AMU:

Farmer 18: *“I had a son there that was in hospital last year and I said if the antibiotics didn’t work for him like we’ll be in trouble and its going that way, so I said I’d try and get involved in it [SDCT] and see does it work”*

There was a more accepting attitude to implementing SDCT when participants acknowledged the benefits to less AMU:

Farmer 16: *“Cows that were on selective dry cow that did get a case of mastitis, it was easier to cure them because there was nothing in their system”*

Farmer 7: *“The massive benefit for me now is in the spring when I don’t have to be watching withdrawal periods and its fantastic that way plus, you’re sending more milk and all that you know so you have the benefit of that as well”*

#### Peer influence

This theme, although also identified as a barrier, was also voiced as a positive influence for encouraging peers into better practice and getting the consult, so advertising SDCT in a helpful manner:

Farmer 4: *“We would look at what way we went last year, and they’d look at doing it and just looking at our downfalls and trying to learn a bit from us”*

Farmers expressed influence from their peers as both negative and positive in regard to adopting SDCT.

## Discussion

To date, there has been limited research exploring what farmers know, think and believe about SDCT, and this is the first study specifically exploring the subject in Ireland. The practice of SDCT is not the current norm in Ireland and represents a significant shift in practice across the industry. This is set against a running clock, as legislative changes accelerate the pace at which the practice of preventative use of antibiotics must cease. This study derived exploratory data highlighting the varied perceptions held by dairy farmers after their dry cow consult. Overall, 6 barriers and 6 facilitators to implementing SDCT on their farms were identified. The results of this study are novel and are imperative in the approach and success of delivery of such a change for the dairy sector.

### Barriers to implementing SDCT

Beliefs in the consequences, and the associated emotional reactions, of moving from BDCT to SDCT had a strong influence on farmers. The fear of increasing mastitis incidence from both farmers who had started SDCT and those who still implemented BDCT was evident, influencing a reluctance towards SDCT. This fear can be expected due to the many economic losses associated with mastitis [24, 25]. The economic impact was highlighted by Geary et al. [26] who estimated that a 4-fold increase in SCC could decrease net farm profits by over 50% on Irish dairy farms. Thus, farmers’ fear of *“taking all the risk”* can be understood as logical. This fear caused many to delay implementing or increasing SDCT which can also be linked to their lack of confidence in the practice. This finding is reflective of previous literature of dairy farmers expressing concerns about implementing SDCT for fear of mastitis, death and unknown financial effects [11, 15].

Farmers’ fear of mastitis could also explain the observed heavy reliance on antibiotics. The dependency on antibiotics as the only perceived defence against mastitis, or *“insurance policy*”, hinders efforts to decrease AMU and changing this mindset towards preventive care instead could prove difficult to overcome. Shortall et al. [27] reported that farmers perceive disease as unavoidable so do not see themselves able to prevent it. Similarly, Swinkels et al., [28] found dairy farmers routinely prolonged their antimicrobial therapy for mastitis due to insecurities in controlling mastitis. The fear factor of increasing mastitis and fear that cows won’t recover without antibiotic dry cow therapy has been seen in many other studies [22] and is a significant challenge to successfully address in increasing the uptake of SDCT.

The social support, such as in the provision of assistance and practical support and advice provided from the farmer’s veterinarian was found to be an important defining construct in the current study. The lack of TASAH-trained veterinarians was found to hinder SDCT implementation as farmers usually picked a geographically convenient practitioner rather than one that they may have an existing relationship with, potentially restricting the effectiveness of the consult. Nonetheless, the lack of preventive advice from veterinarians was highlighted as farmers tend to use them as *“fire brigades”.* Whether there is a lack of farmers seeking preventive advice or if veterinarians are failing to provide or failing to advertise their services as providers of preventive advice, is not clear from the interviews. Literature has correspondingly found dairy farmers to only obtain veterinary advice when disease occurs or after antibiotic administration [18, 29]. Similarly, veterinarians have perceived a lack of demand for preventative services from their clients across various sectors [30]. Veterinarians have also expressed various difficulties in getting engagement in the issue of SDCT, as well as a number of other barriers from their point of view, in the successful implementation of SDCT [8]. Addressing this concern, to facilitate productive engagement between farmers and suitably trained veterinarians, is a hurdle that must be addressed to reduce AMU.

Perceived resource constraints emerged as a key barrier. Limited cubicle space or inadequate housing and infrastructure results in difficulty maintaining high standards of hygiene in order to prevent mastitis over the dry period and was identified as a barrier to uptake of SDCT. A recently published, large scale scoping review outlined farm management issues as a significant barrier to antimicrobial stewardship [31]. A recent Irish report undertaken to identify risks and protective strategies for cow welfare associated with large dairy herd expansion in Ireland reported 32.9% of farmers provided less than 1 cubicle per cow [32]. In addition, Ireland’s seasonal calving system dictates that large numbers of cows are due for dry off at the same time and some herds can dry off all cows before planned start of calving resulting in high stocking rates, potential for housing issues to be exacerbated as well as the added labour involved. Farmers across many countries report cost of production and associated costs of reducing antimicrobials as a potential barrier to uptake [33]. Therefore, this identified barrier, in terms of lack of infrastructure and the cost associated with addressing these concerns, hinders efforts to lower AMU. This fear is particularly heightened as drying off is a particular time when cows are more vulnerable to infection and excellent standards of animal husbandry and correct infrastructure are two key drivers of the success of SDCT.

Dairy farmers have reported their practices to be influenced by financial factors with some studies finding reducing costs the main motive to reduce AMU [18, 22]. However, French dairy farmers reported costs as a constraint to SDCT implementation [15]. Thus, the observed mixed perceptions of the cost of SDCT could be causing confusion amongst farmers and influencing uptake. Negative perceptions of the SDCT workload were found to affect farmers’ attitudes towards it and these negative perceptions need overcome to influence SDCT implementation.

This study agrees with findings from previous literature and has demonstrated dairy farmers to be influenced by their peers [15, 18]. The interviews found farmers to perceive their peers as potential sources of bad habits and peer pressure. Correspondingly, Swinkels et al., [28] found the pressure not to be classed as a ‘bad farmer’ by peers caused farmers not to discuss their mastitis problems. If the benefits of SDCT and the process itself is not universally understood or accepted peer influence has the potential to substantially hinder efforts to reduce AMU.

### Facilitators to implementing SDCT

Self-efficacy was a key psychological construct influencing farmers’ non(engagement) in SDCT. By referring to self-efficacy we refer to an individual’s subjective perception as to his/her ability to perform in a given setting or to attain desired results, proposed as a primary determinant of motivational and emotional states and behavioural change as defined by the APA dictionary of psychology. By undertaking SDCT practices in a graded, “baby step” approach, farmers are able to gradually build confidence that was previously shown to be lacking, influencing a fear of SDCT and reliance on antibiotics; so, this facilitator could help to overcome these barriers. Correspondingly, conducting small trials of SDCT to build trust slowly was cited by veterinarians as a way to facilitate SDCT introduction [8]. Farmers need self-confidence to implement changes [34, 35]. Therefore, promoting this steady approach should be encouraged by veterinarians to help increase SDCT uptake. Having a positive and helpful consult was identified as a facilitator towards implementing SDCT. This was expected as literature has cited the positive influence a veterinarian can have on dairy farmers’ AMU and AMR awareness through frequent contact [10, 22]). Veterinary guidance is influential as there is abundant literature reporting how farmers trust and value it [20, 36]. Furthermore, veterinary advice has been cited as the most effective approach to influence farmers to implement SDCT [37]. Getting along well with the veterinarian and feeling they improved practice was significant in the farmers’ perceived outcome of the consult, which suggests the veterinarians’ approach was successful. More effective consults took place when both the farmer and veterinarian had input. Equally, Scherpenzeel et al., [37] reported mutual communication is needed for changing behaviour successfully. Moreover, the consult allowed for specific advice to be given which was positively reported in the farmers’ feedback, reflecting literature that advice tailored to the farmers’ needs is more effective for motivating farmers [38]. The cited need for veterinary input in the future is similar to findings from Swiss and Dutch farmers who reported the need for veterinary support in future herd health control programmes [34, 39]. Higgins et al. [8] found veterinarians to acknowledge a need for more discussions with their farmer clients about SDCT. Both the interviews in this study, and published literature, emphasize the importance of the veterinarian for future implementation of SDCT, which could also be utilised to increase the promotion of veterinary preventive advice in general.

The need for high standards of hygiene and husbandry to prevent mastitis are widely accepted as a key to mastitis control. Positively, the importance of hygiene was cited frequently in the interviews, despite literature finding dairy farmers to lack awareness or appreciation of the importance of implementing preventive hygiene measures [40]. Successful implementation of this facilitator, and promotion of strategies to address hygiene related concerns, could help to alleviate some barriers to SDCT. In order to correctly select cows for antibiotic treatment, farmers need to be milk recording regularly, which was frequently acknowledged in the interviews, highlighting its key role in facilitating the practice of SDCT. This is a key challenge in Ireland as currently, approximately 40 % of herds in Ireland are milk recording [41].

The main reason farmers cited for getting involved with the TASAH dry cow consult in this study was that they knew SDCT was becoming compulsory and mixed attitudes were displayed towards the upcoming introduction of SDCT. Regulatory changes are noted as key measure required to succeed in achieving behaviour change in other countries such as The Netherlands [23]. Preventive use of antimicrobials was prohibited in The Netherlands in 2012, causing the increased implementation of SDCT by 2013, reducing dry cow antibiotics by approximately 28% [42]. Surveyed British veterinarians stated the need for external regulatory pressures to change dairy farmers’ behaviour from BDCT to SDCT [8]. In Ireland the positive effect of regulatory pressure has been witnessed in other disease control programs, such as the national BVD eradication program. Consequently, despite some barriers, legislation triggers mass changes to dairy farmers’ behaviour and in Ireland this can be presumed to have the same effect.

Farmers that acknowledge AMR as an emerging health issue will be integral to the acceptance and delivery of SDCT across Ireland. The interviews in this study highlighted that farmers that have knowledge and awareness of the benefits to reduced AMU, for the greater good of human and animal health, also had a more positive outlook on SDCT. Studies have found a high awareness of AMR amongst dairy farmers [10, 18]. However, as AMR was not commonly cited in this study, it suggests AMR is not as well known or perceived as a problem in Ireland. Correspondingly, studies have found reluctance amongst dairy farmers to take responsibility for the growth of AMR or it was not recognised by them as a reason to reduce AMU [20, 28]. Furthermore, veterinarians have cited the difficulty in engaging with farmers when they do not perceive AMR as a problem [8], thus hindering efforts to introduce SDCT. To influence farmers’ behaviour, they need to recognise and accept their responsibility in the existence of the problem [38]. This could explain why a certain population of farmers were resistant to reducing their AMU and felt it was acceptable to rely heavily on antibiotics. Educating farmers on the threat of AMR and the benefits of reducing AMU could be utilised to help farmers acknowledge the issue. British veterinarians have previously cited this education drive as beneficial [8, 11] and reported that, after receiving training on SDCT, British dairy farmers felt more prepared and would recommend it to other farmers. Therefore, if the education of farmers on the context of why SDCT is important in the fight against AMR was more widespread across the whole country, this could help farmers to overcome the fear of SDCT. In turn this will also help decrease reliance on antibiotics, clarify the mixed perceptions of SDCT, and increase confidence in the practice. There is an urgent need to consider these contributing factors to facilitate the move towards SDCT in Ireland. This study highlights the need for a collaboration between the social science and agricultural science disciplines given the complexities identified in changing to SDCT. The overwhelming need for collaboration across the sector is highlighted as fundamental to successful delivery of such a cultural change as SDCT in Ireland.

As already discussed, this study demonstrated dairy farmers to be influenced by their peers, however this influence was found to be both potentially negative and positive. This study found peer influence to have a potentially facilitating influence by encouraging farmers and promoting SDCT. Likewise, literature has found peer influence to be a main driver towards reducing AMU [18]. Also, ‘hearsay’ amongst farmers has been reported as strong proof a certain practice is beneficial [8]. Therefore, the potential barriers caused by peer influence may be overcome by tactically using it to facilitate more prudent AMU and the move towards SDCT.

A number of opportunities could be developed from this study, the first to explore farmer beliefs about SDCT in Ireland. The facilitators that have emerged could be used to drive innovation and provide lessons in how to address the significant challenge required to adopt new practices, that are currently at odds with what is culturally and habitually ingrained in many Irish farmers. Perhaps, seizing the opportunity to engage with the innovators and early adopters of SDCT and use their experiences could be a beacon of change, where farmers learn from other farmers. Education and communication campaigns are necessary to disseminate the truths about SDCT and the farmers’ role in addressing AMR. Further, the role of trained vets in providing a valued service in advice and training in the area of SDCT needs to be further developed and promoted.

There are a number of limitations to this study. Whilst the recruitment approach captured a range of farmers, the nature of the study did not make allowance for direct comparisons between age or farm size which could influence attitudes towards AMU. As sign up to the consult was voluntary with eligibility criteria, this could introduce potential bias as it would include farmers already with an interest in gaining dry cow therapy advice. Only male farmers are represented, which is a further limitation to the representativeness of the participants. There are not enough interviews to draw inference form differing attitudes by farm type, size, location or other explanatory variables.

## Conclusion

These findings point to a number of important psychological constructs which can be leveraged in future interventions aimed at supporting behaviour change specific to SDCT in the Irish dairy sector. These constructs include self-efficacy, beliefs in consequences, emotions, perceived resource constraints, social and cultural norms and social support. A significant barrier identified was the fear of increasing mastitis incidence amongst farmers which led to a reluctance towards SDCT. This fear was suggested to be linked to farmers’ heavy reliance on antibiotics to control mastitis. A key barrier determined was the limited utilisation of preventive herd health advice. Lacking infrastructure and mixed perceptions of SDCT and peer influence were also found to hinder SDCT implementation. However, it became evident the facilitators offer solutions to overcome the barriers. Implementing SDCT using a slow but steady approach will gradually build farmers’ confidence in reducing AMU and can help to overcome the fear of SDCT and reliance on antibiotics. More widespread use of targeted veterinary consults and herd management advice that engages farmers and veterinarians in a discussion about disease prevention measures was highlighted as vital for successful SDCT implementation. This engagement could also help prioritise and address weakness’ in infrastructure that may facilitate farmers being more comfortable with SDCT. Regulatory pressure was the most cited reason to start SDCT. Nonetheless, widespread education of farmers on the real threat of AMR and their role and responsibility in reducing AMU by the practice of SDCT is recommended to increase confidence, overcome fear of SDCT and reliance on antibiotics, and explain any regulatory change. Peer influence has a powerful impact and should be used more effectively to educate farmers on SDCT and help hasten the successful transition towards more widespread use of SDCT in Ireland. This novel study investigating the facilitators and barriers which are shaping farmer engagement with SDCT in Ireland is timely given the backdrop of the new regulations. Further, it highlights the pivotal role of social and behavioural science in agriculture and the urgent need for a collaborative, interdisciplinary approach that will be required to effectively deliver what is a momentous change in practice in the dairy sector. The facilitators determined from this study could be incorporated into SDCT programmes and used to inform education and communication drives across the country in Ireland to promote the practice and ensure a safe move towards SDCT, while the barriers will also need to be targeted through behaviour change strategies.

## Methods

### Study context and participants

As part of the Rural Development Plan 2014-2020, the Irish Department of Agriculture Food and the Marine (DAFM), in conjunction with the EU, are funding a Targeted Advisory Service on Animal Health (TASAH) for farmers, delivered by trained veterinary practitioners. Animal Health Ireland, which is a not-for-profit organisation, is responsible for the delivery of training to practitioners and coordination of the service to farmers. As part of this, the CellCheck programme developed specific training for veterinarians to deliver dry cow consults with their farmer clients, on maximising dry period udder health performance and moving safely towards SDCT. In addition, the funding also provides for farmers to avail of a TASAH-funded dry cow consult with a trained veterinary practitioner. The purpose of this consult is to enable farmers to engage with a suitably trained veterinarian to develop farm specific selective dry cow strategies where appropriate, specifically advising how to move safely towards SDCT. Several eligibility criteria applied for farmers to avail of this funded dry cow consult in its initial pilot year 2018. These criteria included a consistently low bulk milk somatic cell count (< 200,000 cells/ml) in the previous 12 months, regular milk recording including within a defined period pre-dry off, as well as being willing to be involved in feedback and data collection [14]. Part of the criteria for availing of the funded consult included consent to be interviewed after the subsequent calving season, to evaluate the experience of their process. The aim of the interview was to understand farmers’ barriers and facilitators of SDCT and if they had chosen to implement SDCT based on the experience in the consult. The study participants were farmers who voluntarily applied for the dry cow consult in Autumn 2018, completed the consult and the follow up interviews the following summer and hence they were a convenience sample. Nineteen participant farmers were used for this study. The participants were all male, spring calving farmers, located throughout Ireland. The mean herd size of participants was 116 cows (range 60-346). The Irish average dairy herd size is 90 cows [43]. The participants supplied 6 different processors (of which there are 12 in Ireland) and were located throughout Ireland, participants represented 10 counties.

### Interviews

All dry cow consults between participating farmers and the trained veterinary practitioners took place in Autumn 2018. All farmers were contacted by telephone by a final year veterinary student, the second author, as part of a summer research project in July and August 2019 subsequent to the conclusion of the spring 2019 calving season. The aim was to gain as much information as possible about whether participating farmers had adopted changes to their drying off methods after the consult, if they perceived these changes to have had any impact on mastitis the following calving season and whether this impacted their attitudes to SDCT. A semi-structured style of questioning was adopted to conduct the interviews. A copy of the questionnaire is included as supplementary material.

### Data analysis

Audio recordings were professionally transcribed verbatim. The transcripts were coded using an inductive thematic analysis procedure as outlined by Braun and Clarke [44] in order to identify emerging themes. Firstly, transcripts were read over to gauge the topics covered and generate ideas. Any significant statement made towards SDCT was assigned a code. This process was repeated to ensure intra-code reliability. The transcripts were independently coded by another researcher. These codes were compared, and a joint consensus was reached on the application of codes to ensure inter-coder reliability. It was decided that data saturation had occurred as no new codes emerged from the final 5 interviews. Similar codes were then grouped together to make 12 potential themes. These themes were inspected and refined to ensure they were distinct and clearly communicated the interview results. The themes were then separated as barriers or facilitators to SDCT. To ensure thoroughness, the transcripts were re-read again to check no significant data had been missed from previous coding stages. Finally, relevant quotes were selected from the transcripts as examples to exemplify each barrier and facilitator.

## Supplementary Information

**Additional file 1.** Selective Dry Cow Therapy TASAH Follow-up Questionnaire.

## Data Availability

The datasets used and/or analysed during the current study are available from the corresponding author on reasonable request.

## Abbreviations

SDCT: Selective dry cow therapy
AMR: Antimicrobial resistance
WHO: World Health Organization
BDCT: Blanket dry cow therapy
AMU: Antimicrobial usage
SCC: Somatic cell count
DAFM: Irish Department of Agriculture Food and the Marine
TASAH: Targeted Advisory Service on Animal Health
