# Supplementary material for: Engaging with selective dry cow therapy: understanding the barriers and facilitators perceived by Irish farmers
Source: Ir Vet J. 2021 Oct 23;74:28. doi: 10.1186/s13620-021-00207-0 (PMC8540178; doi:10.1186/s13620-021-00207-0)
Supplement: Supplementary file 1 — Additional file 1. Selective Dry Cow Therapy TASAH Follow-up Questionnaire. [file 13620_2021_207_MOESM1_ESM.docx]

Selective Dry Cow Therapy TASAH Follow-up Questionnaire

FARMER VERSION

Farmer name:

Herd No:

Vet info:

**Section 1 – The Consult**

1. **Talk me through how and why you got involved in the TASAH Selective Dry Cow Therapy?**

________________________________________________________________________________________________________________________________________________________________________________________________________________________________________________________________________________________

1. **What were the recommendations given to you by the vet during the consult?**

___________________________________________________________________________________________________________________________________________________________________________________________________________________________________________________________________________

1. **What do you feel was the most useful recommendation that was given to you and why?**

____________________________________________________________________________________________________________________________________________________________________________________________________________________________________________________________________________

1. **What do you feel went well with the consult with the vet?**

____________________________________________________________________________________________________________________________________________________________________________________________________________________________________________________________________________

1. **Is there anything you feel could be improved on in the consult?**

____________________________________________________________________________________________________________________________________________________________________________________________________________________________________________________________________________

1. **How did you choose your vet?**

____________________________________________________________________________________________________________________________________________________________________________________________________________________________________________________________________________

1. **Has the consult vet followed up with you?**

____________________________________________________________________________________________________________________________________________________________________________________________________________________________________________________________________________

1. **Have you discussed mastitis, SCC or milk quality with your vet in spring 2019?**

____________________________________________________________________________________________________________________________________________________________________________________________________________________________________________________________________________

1. **Did the consult highlight any other problem areas on the farm i.e. were there any unrelated benefits to doing the SDCT consult?**

____________________________________________________________________________________________________________________________________________________________________________________________________________________________________________________________________________

1. **Did you feel the consult was worthwhile overall?**

____________________________________________________________________________________________________________________________________________________________________________________________________________________________________________________________________________

**Section 2 – What the farmer did**

1. **What did you do on your farm following the visit by the vet?**

____________________________________________________________________________________________________________________________________________________________________________________________________________________________________________________________________________

1. **If you carried out the SDCT, what exactly did you do?**

____________________________________________________________________________________________________________________________________________________________________________________________________________________________________________________________________________

1. **How many cows did not get an antibiotic at drying off?**

____________________________________________________________________________________________________________________________________________________________________________________________________________________________________________________________________________

1. **Out of these cows how many got sealer?**

____________________________________________________________________________________________________________________________________________________________________________________________________________________________________________________________________________

1. **How many of these cows got nothing?**

____________________________________________________________________________________________________________________________________________________________________________________________________________________________________________________________________________

1. **For the cattle that received an antibiotic, what antibiotic did they get?**

____________________________________________________________________________________________________________________________________________________________________________________________________________________________________________________________________________

1. **Did the cows that got antibiotic get a teat sealer also?**

____________________________________________________________________________________________________________________________________________________________________________________________________________________________________________________________________________

1. **How do you feel the SDCT went?**

____________________________________________________________________________________________________________________________________________________________________________________________________________________________________________________________________________

1. **If you didn’t carry out the SDCT, what did you do instead?**

____________________________________________________________________________________________________________________________________________________________________________________________________________________________________________________________________________

1. **Why didn’t you carry out the SDCT?**

____________________________________________________________________________________________________________________________________________________________________________________________________________________________________________________________________________

1. **Have you milk recorded this year?**

____________________________________________________________________________________________________________________________________________________________________________________________________________________________________________________________________________

1. **If yes, when was the first recording?**

____________________________________________________________________________________________________________________________________________________________________________________________________________________________________________________________________________

**Section 3 – The Future**

1. **Will you be carrying out SDCT this year?**

____________________________________________________________________________________________________________________________________________________________________________________________________________________________________________________________________________

1. **Is there anything you would do differently next year that you learned from either the consult with the vet or the process of carrying out the SDCT?**

____________________________________________________________________________________________________________________________________________________________________________________________________________________________________________________________________________

1. **Would you recommend this process to other farmers?**

____________________________________________________________________________________________________________________________________________________________________________________________________________________________________________________________________________

1. **How do you feel about not using dry cow antibiotic in cows that don’t have any sign of infection?**

____________________________________________________________________________________________________________________________________________________________________________________________________________________________________________________________________________
